# Noncovalent Protein–Pseudorotaxane Assembly Incorporating an Extended Arm Calix[8]arene with α-Helical Recognition Properties

**DOI:** 10.1021/acs.cgd.0c01717

**Published:** 2021-02-08

**Authors:** Niamh M. Mockler, Kiefer O. Ramberg, Francesca Guagnini, Colin L. Raston, Peter B. Crowley

**Affiliations:** † School of Chemistry, National University of Ireland Galway, University Road, Galway, H91 TK33, Ireland; ‡ Flinders Institute for Nanoscale Science and Technology, College of Science and Engineering, Flinders University, Bedford Park, South 5042, Australia

## Abstract

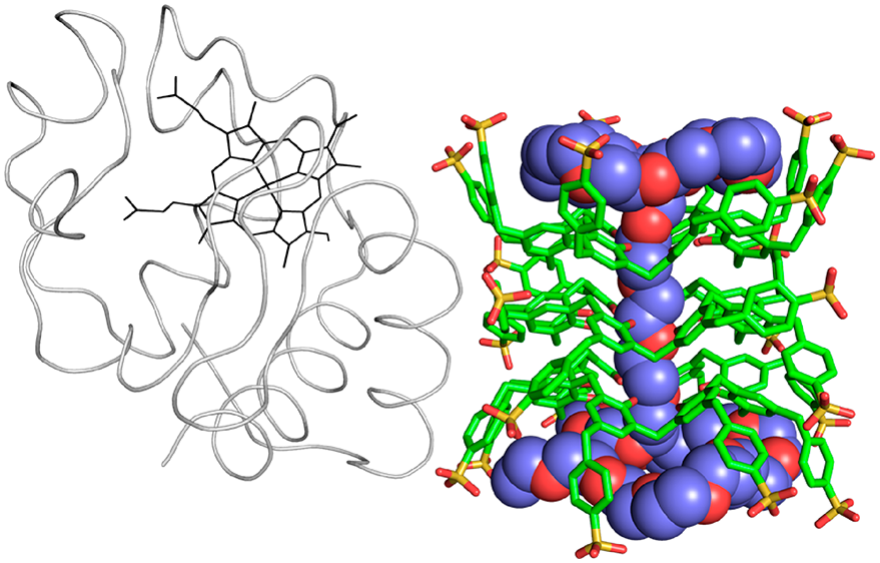

Water-soluble, anionic calix[*n*]arenes are useful receptors for protein recognition and assembly. For example, sulfonato-calix[8]arene (**sclx**_**8**_) can encapsulate proteins and direct their assembly into porous frameworks. In this work, we turned our attention to an “extended arm” calixarene with 16 phenyl rings. We hypothesized that this larger receptor would have increased capacity for protein masking/encapsulation. A cocrystal structure of *p*-benzyl-sulfonato-calix[8]arene (**b-sclx**_**8**_) and cytochrome *c* (cyt *c*) revealed a surprising assembly. A pseudorotaxane comprising a stack of three **b-sclx**_**8**_ molecules threaded by polyethylene glycol (PEG) was bound to the protein. The trimeric **b-sclx**_**8**_ stack, a tubelike structure with a highly charged surface, mediated assembly via a new mode of protein recognition. The calixarene stack presents four hydrophobic grooves, each of which binds to one cyt *c* by accommodating the N-terminal α-helix. This unprecedented binding mode suggests new possibilities for supramolecular protein chemistry.

## Abstract 2

Extended arm calix[8]arene self-assembles as a trimeric stack with hydrophobic grooves that accommodate an α-helix of cytochrome *c*. The calixarene stack is threaded by polyethylene to yield a pseudorotaxane.

The complex topology of protein surfaces presents ample opportunity for recognition by synthetic molecules, resulting in diverse functions.^1−4^ Small molecules that occupy α-helical-binding grooves on a protein surface can be applied to inhibit protein–protein interactions.^1^ Supramolecular receptors, such as cucurbit[*n*]urils^5−8^ and calix[*n*]arenes,^9−20^ that target specific side chains have applications in controlled protein assembly and noncovalent PEGylation of biopharmaceuticals. Water-soluble, anionic calixarenes have useful recognition properties arising from their affinity for cationic residues (Lys, Arg). The commercially available sulfonato-calix[*n*]arenes (**sclx**_***n***_) are gaining traction as off-the-shelf mediators of protein assembly. Acting as “molecular glues”, **sclx**_***n***_ can mediate protein oligomerization and direct the packing of protein frameworks.^14,16,18,20^ Such frameworks provide a foundation for biodegradable and biocompatible materials with potential applications in drug delivery, catalysis, protein-based coatings, and more.^2,3,21^ Additionally, calixarenes can be employed in mechanically interlocked molecules (MIMs), including rotaxanes and catenanes.^22−26^ Consequently, there is opportunity to generate MIMs with protein recognition capacity, providing new types of biohybrid materials and/or molecular machines. Here, we describe the protein recognition and assembly activity of *p*-benzyl-sulfonato-calix[8]arene (**b-sclx**_**8**_, Figure 1), an extended arm calixarene.^27^

**Figure 1:**
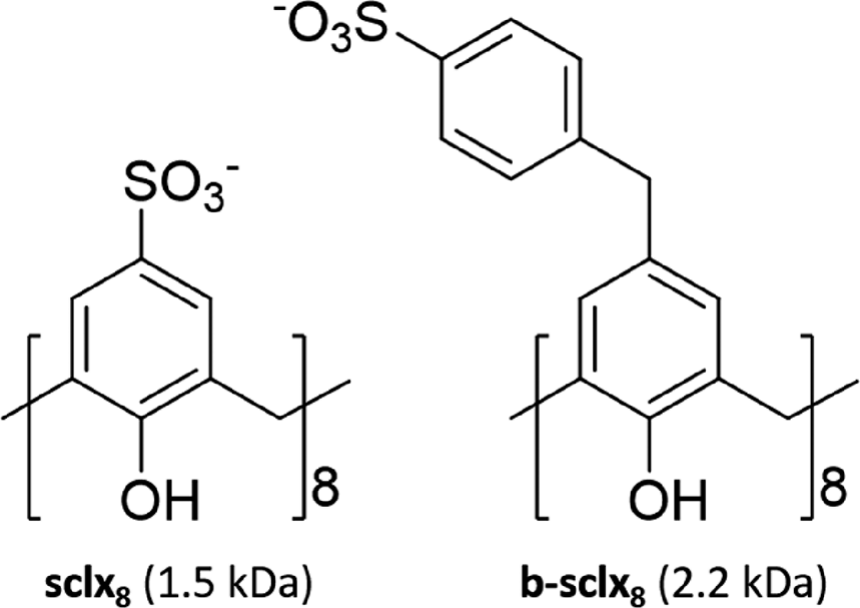
Sulfonato-calix[8]arene (**sclx**_**8**_) and *p*-benzyl-sulfonato-calix[8]arene (**b-sclx**_**8**_).

Crystal structures of cationic proteins in complex with differently functionalized calix[4]arenes,^9,12,13,15,16,19^ calix[6]arenes,^11,16,17^ and calix[8]arenes^14,16,18^ have highlighted the advantages of larger calixarene hosts. The inherent flexibility of **sclx**_**6**_ and **sclx**_**8**_ compared to the bowl-shaped **sclx**_**4**_ facilitates topological molding to protein surfaces and greater surface coverage. Our recently reported crystal structures have established that **sclx**_**8**_ (Figure 1) can mask up to ∼30% of the cytochrome *c* (cyt *c*) surface.^14^ Autoregulated assembly of cyt *c* was achieved, with tetramerization at 1 equiv and oligomer disassembly at ≥3 equiv of **sclx**_**8**_.^14^ Furthermore, crystalline frameworks of cyt *c* and **sclx**_**8**_ with varying porosities and up to ∼5 nm pore diameter were obtained, as a function of the calixarene concentration.^14,18^ Cyt *c*-**sclx**_**8**_ frameworks were amenable to crystal engineering, with the introduction of a small molecule effector facilitating framework duplication.^18^ Considering these results, we were interested to test how a larger **sclx**_**8**_ derivative, **b-sclx**_**8**_, might manipulate the assembly of cyt *c*. With a solvent accessible surface area (ASA) of 2400 Å^2^, **b-sclx**_**8**_ was expected to mask a larger portion of cyt *c* than **sclx**_**8**_ (ASA = 1600 Å^2^). Complex formation between **b-sclx**_**8**_ and cyt *c* was studied by NMR spectroscopy and X-ray crystallography, the latter revealing an unexpected biohybrid assembly.

Initially, we investigated the interactions of cyt *c* and **b-sclx**_**8**_ via ^1^H–^15^N HSQC-monitored titrations in 20 mM sodium acetate, 50 mM NaCl at pH 5.6. Backbone amide chemical shift perturbations were observed at 2 equiv of **b-sclx**_**8**_. The resonances of Ala3, Lys4, Lys5, Thr12, and Lys86 were perturbed, suggesting that **b-sclx**_**8**_ interacted with part of the known calixarene binding patch on cyt *c* (Figure 2).^9,11−15,17−19^ At ≥ 4 equiv of **b-sclx**_**8**_, the sample precipitated, resulting in signal loss in the ^1^H–^15^N HSQC spectrum and precluding further analysis. Therefore, we turned to crystallography to investigate the protein–calixarene interaction in more detail.

**Figure 2:**
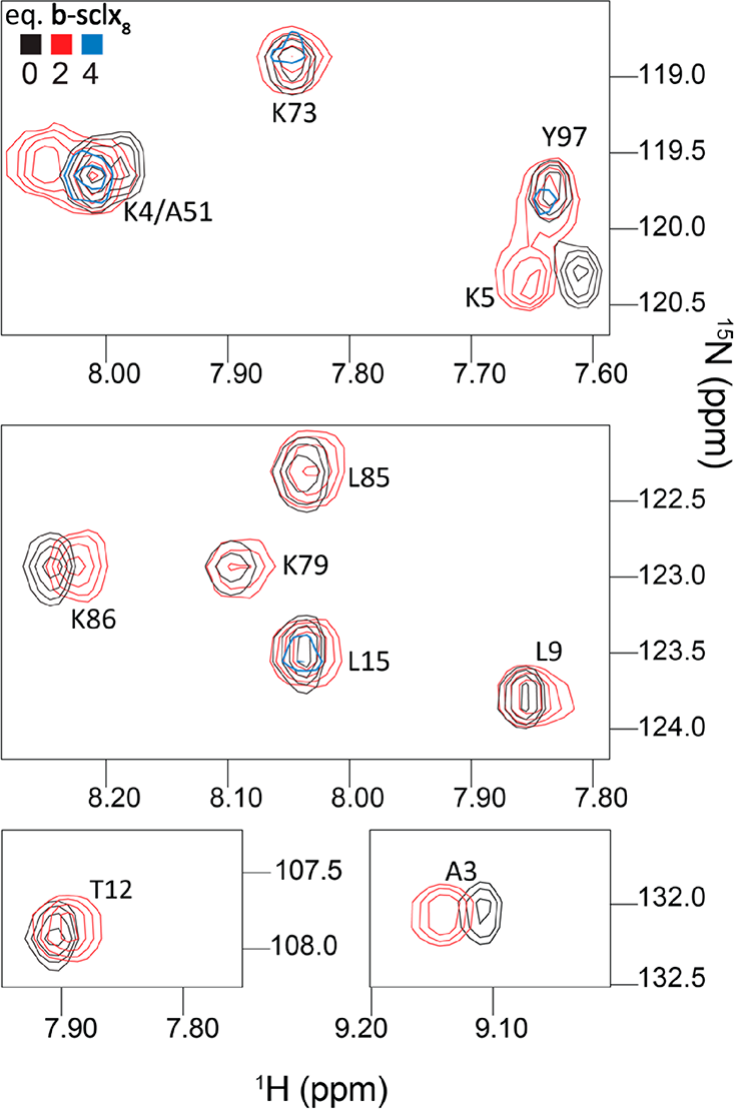
Overlaid regions of the ^1^H–^15^N HSQC spectra of cyt *c* in the absence (black contours) and presence of 2 (red) or 4 (blue) equiv of **b-sclx**_**8**_, in 20 mM sodium acetate and 50 mM NaCl, pH 5.6. Note the signal loss at 4 equiv of **b-sclx**_**8**_ due to sample precipitation.

Cocrystals of cyt *c* and **b-sclx**_**8**_ were grown by sitting drop vapor diffusion using a sparse matrix screen (Jena JCSG++ HTS).^14^ Small crystals (<40 μm dimension) appeared in eight conditions of the screen. In addition to crystals, a brown precipitate formed at 2–4 equiv of **b-sclx**_**8**_ (Supporting Information, Figure S1). The crystallization conditions contained 20% PEG 3350 or 30% PEG 2000 and 100–200 mM salts including potassium formate, magnesium formate, ammonium formate, or ammonium chloride. Crystals were reproduced manually in similar conditions. **b-sclx**_**8**_ was necessary for crystallization as crystals did not grow in the absence of the macrocycle. Diffraction data were collected at SOLEIL synchrotron to 3.0 Å resolution. Several crystals were tested on two occasions, with no further improvement in resolution. The crystal structure was solved by molecular replacement in space group *C*121, with four cyt *c* molecules in the asymmetric unit (Table S1). The presence of **b-sclx**_**8**_ was evident in the unbiased electron density maps (Figure S2), and a stack of three **b-sclx**_**8**_ was modeled. A tube of electron density running through the center of the calixarene stack was modeled as PEG (*vide infra*), resulting from the use of PEG as the precipitant.

In the crystal structure (PDB entry 7BBT), the **b-sclx**_**8**_ molecules adopt the fully extended, pleated loop conformation. The trimeric stack is ∼6.6 kDa (approximately half the mass of cyt *c*), ∼2.1 nm long and held together via CH−π and π–π interactions (Figure 3). In the central calixarene, the methylene bridges form CH−π bonds alternately to phenyl rings above or below in the stack. The benzyl-sulfonato arms project outward from the stack for the outer calixarenes, and point up/down for alternate substituents in the central calixarene. As a consequence of the stacking and the projections of the extended arms, the **b-sclx**_**8**_ trimer presents four hydrophobic grooves. Each groove accommodates the N-terminal α-helix of one cyt *c* by binding the methyl substituents of Thr8 and Thr12 (Figure 3). The slotting of the α-helix into the **b-sclx**_**8**_ groove is complemented by characteristic protein–calixarene charge–charge interactions. The side chain of Thr8 is flanked by Lys4, Lys5, and Lys11 each of which interacts with a sulfonate ion. The Thr12 side chain is flanked by Lys11 and Arg13, the latter forms a cation−π bond to **b-sclx**_**8**_. Thus, in contrast to **sclx**_**8**_,^14,16,18,20^ the conformation of **b-sclx**_**8**_ was not altered to “fit” the protein surface, and encapsulation was not achieved. Nevertheless, the trimeric stack presents a binding patch with a hydrophobic core, and a charged periphery that complements the oppositely charged protein surface. A comparison of the NMR data (Figure 2) and the crystal structure (Figure 3) indicates similarities and differences. For example, the NMR suggests binding at Lys4 and Lys5 but not at Arg13.

**Figure 3:**
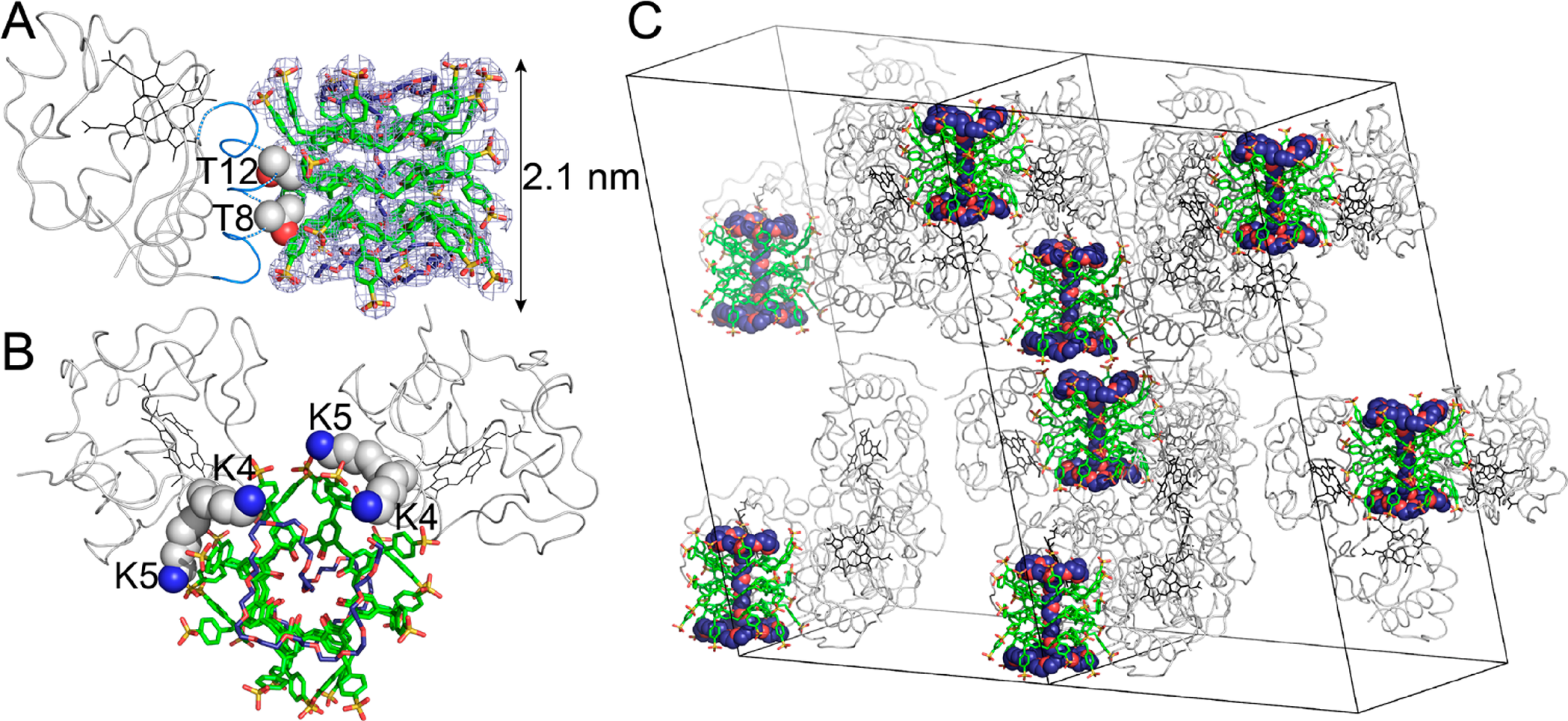
(A) The N-terminal α-helix of cyt *c* inserts into a hydrophobic groove presented by a trimeric stack of **b-sclx**_**8**_. The stack is threaded by PEG (navy) giving rise to pseudorotaxane. The 2Fo-Fc electron density map (contoured at 0.5σ) is shown as a blue mesh. (B) Lys4 and Lys5 interact with the calixarene stack via salt bridges to the sulfonate ions. (C) Crystal packing in the cyt *c* and **b-sclx**_**8**_ structure. Adjacent pairs of **b-sclx**_**8**_ stacks are in close proximity (<6 Å). Axes denote the unit cells.

Surprisingly, the crystal structure included a PEG fragment of 29 monomers (1.3 kDa) threaded through the cavity of the trimeric **b-sclx**_**8**_ stack, resulting in a [4]pseudorotaxane (Figures 3 and S3). The polyether chain forms multiple van der Waals contacts with all 24 of the phenolic hydroxyls. In addition to threading the cavity, the PEG is laced via CH−π bonds around the benzyl arms projecting away from either end of the stack. There are several examples of calixarene-based pseudorotaxanes reported in the literature, including one with PEG.^22,23,25,26^

Simultaneous interaction of the stacked **b-sclx**_**8**_ with cyt *c* and PEG resulted in a multicomponent pseudorotaxane–protein assembly (Figure 3). Evidently, intermolecular CH−π and π–π stacking interactions between **b-sclx**_**8**_ were favored over calixarene–protein interactions and induced the formation of the calixarene stack. Similar calixarene–calixarene interactions led to a calix[6]arene dimer in a complex with cyt *c*,^11^ and a calix[8]arene dimer in complex with a lectin.^20^ Steric hindrance by the bulky extended arms may have reduced the flexibility of **b-sclx**_**8**_ and decreased the capacity for calixarene molding to the protein surface. The stacking of **b-sclx**_**8**_ resulted in a new type of protein recognition, whereby the trimeric stack presents hydrophobic grooves that can bind an α-helix. Previously, helix mimetics (including foldamers) have been used to slot into helix-accommodating grooves on the protein surface.^1,28^ Here, the calixarene stack presents the groove to accommodate the protein.

In parallel to protein recognition, the **b-sclx**_**8**_ stack facilitated pseudorotaxane formation via PEG threading, adding to the collection of calix[8]arene-based rotaxanes reported previously.^22,23^ The combination of synthetic machines (rotaxanes) and biological machines (proteins) is an innovative, but underdeveloped topic. In the first reported protein–rotaxane conjugate, a [2]rotaxane was generated via covalent linkage of a pseudorotaxane to cyt *c.*(29) Previously, we reported crystal structures in which small PEG fragments were trapped by calix[4]arene or calix[8]arene.^10,15,16^ The pseudorotaxane of **b-sclx**_**8**_ and PEG reported here raises the possibility of new assembly strategies with PEGylated therapeutics.

Finally, the α-helix binding capacity of **b-sclx**_**8**_ mediated the construction of a novel biohybrid material. The structure is comparable to a recently reported foldamer-cyt *c* assembly, in which the protein was bound to cylindrical foldamer stacks, of similar size and shape to the pseudorotaxane.^30^ Comparison can be made also to protein–cucurbituril cluster assemblies with high macrocycle/protein mass ratios.^7^ These biohybrid materials are of interest in crystal engineering, with scope for the inclusion of another biomolecule (protein or peptide) in the assembly. Further research is necessary to investigate the possibilities arising from this novel biohybrid assembly, in particular, the α-helix recognition capacity of the trimeric **b-sclx**_**8**_ stack.

## Supporting Information Available

Experimental details, electron density maps and X-ray data collection, processing, and refinement statistics (PDF)
