# Supplementary material for: Noncovalent Protein–Pseudorotaxane Assembly Incorporating an Extended Arm Calix[8]arene with α-Helical Recognition Properties
Source: Cryst Growth Des. 2021 Feb 8;21(3):1424–7. doi: 10.1021/acs.cgd.0c01717 (PMC8154262; doi:10.1021/acs.cgd.0c01717)
Supplement: Supplementary file 1 — cg0c01717_si_001.pdf [file cg0c01717_si_001.pdf]

Supporting Information

Noncovalent Protein – Pseudorotaxane Assembly Incorporating an Extended Arm Calix[8]arene with α-helical Recognition Properties

Niamh M. Mockler,[a] Kiefer O. Ramberg,[a] Francesca Guagnini,[a] Colin L. Raston,[b] and Peter B. Crowley*[,a]

[a]School of Chemistry, National University of Ireland Galway, University Road, Galway, H91 TK33, Ireland.

[b]Flinders Institute for Nanoscale Science and Technology, College of Science and Engineering, Flinders University, Bedford Park, SA 5042, Australia.

*Correspondence to: peter.crowley@nuigalway.ie, +353 91 49 24 80

Keywords: alpha helix, biomaterials, calixarene, molecular recognition, supramolecular

Experimental

Sample preparation. ^{15}N-labeled and unlabelled *Saccharomyces cerevisiae* cytochrome *c* (cyt *c*, C102T) were expressed in *Escherichia coli* BL21, isolated and purified according to published methods.[1-3] The *p*-benzyl-sulfonato-calix[8]arene (**b-sclx$_8$**) stock solution (40 mM) was prepared in water.[4]

NMR spectroscopy. A 600 MHz Varian spectrometer equipped with a HCN cold probe was used to perform ^{1}H–^{15}N HSQC-monitored titrations at 30 °C, as described previously.[3] NMR samples contained 0.2 mM fully oxidised ^{15}N labelled cyt *c* in 20 mM sodium acetate, 50 mM sodium chloride and 10 % D_2O at pH 5.6. Microlitre aliquots of a 40 mM **b-sclx$_8$** stock were titrated against the cyt *c* sample. Spectra were processed in NMRPipe,[5] and analysed in CCPN.[6]

Co-crystallization of cyt *c* and b-sclx$_8$. An Oryx 8 robot (Douglas Instruments) and the JCSG++ HTS sparse matrix screen (Jena Bioscience) were used to obtain crystals via sitting drop vapour diffusion. Trials were performed on protein-calixarene mixtures (prepared in water) with 0.3-30 eq. **b-sclx$_8$**. Jena conditions A5, A8, A9, A10, A12, G8, G9 and G10, containing 20-30 % PEG 2000-3350 and 100-200 mM salt, yielded red crystals amid brown precipitate. Crystals were reproduced manually via hanging drop vapour diffusion in 24 well Greiner plates. Crystals were cryo-protected in their respective reservoir solution supplemented with 25% glycerol and cryo-cooled in liquid nitrogen.

X-ray Data Collection and Structure Determination. Diffraction data were collected to 3.0 Å resolution at beamline Proxima 2A (SOLEIL synchrotron). Diffraction frames were processed using the autoPROC pipeline,[7] with integration in XDS,[8] scaling and merging in AIMLESS[9] and POINTLESS.[10] The structure was solved by molecular replacement in PHASER[11] using one molecule of cyt *c* (PDB 6RSL) as the search model.[12] Identical results were obtained for two crystals on two separate occasions. Coordinates and restraints for **b-sclx$_8$** were generated in the Mercury software[13] and GRADE Web Server.[14] Model building in COOT[15] and refinement in PHENIX[16] and BUSTER[17] were continued iteratively until the electron density and R_{free} could be improved no further.[18] Refinement statistics are reported in Table S1. The structure and associated structure factor amplitude were deposited in the Protein Data Bank under the code 7BBT (Table S1) after validation in MolProbity.[19] Accessible surface areas (ASA) were analysed in AreaIMol (CCP4 suite).[20]

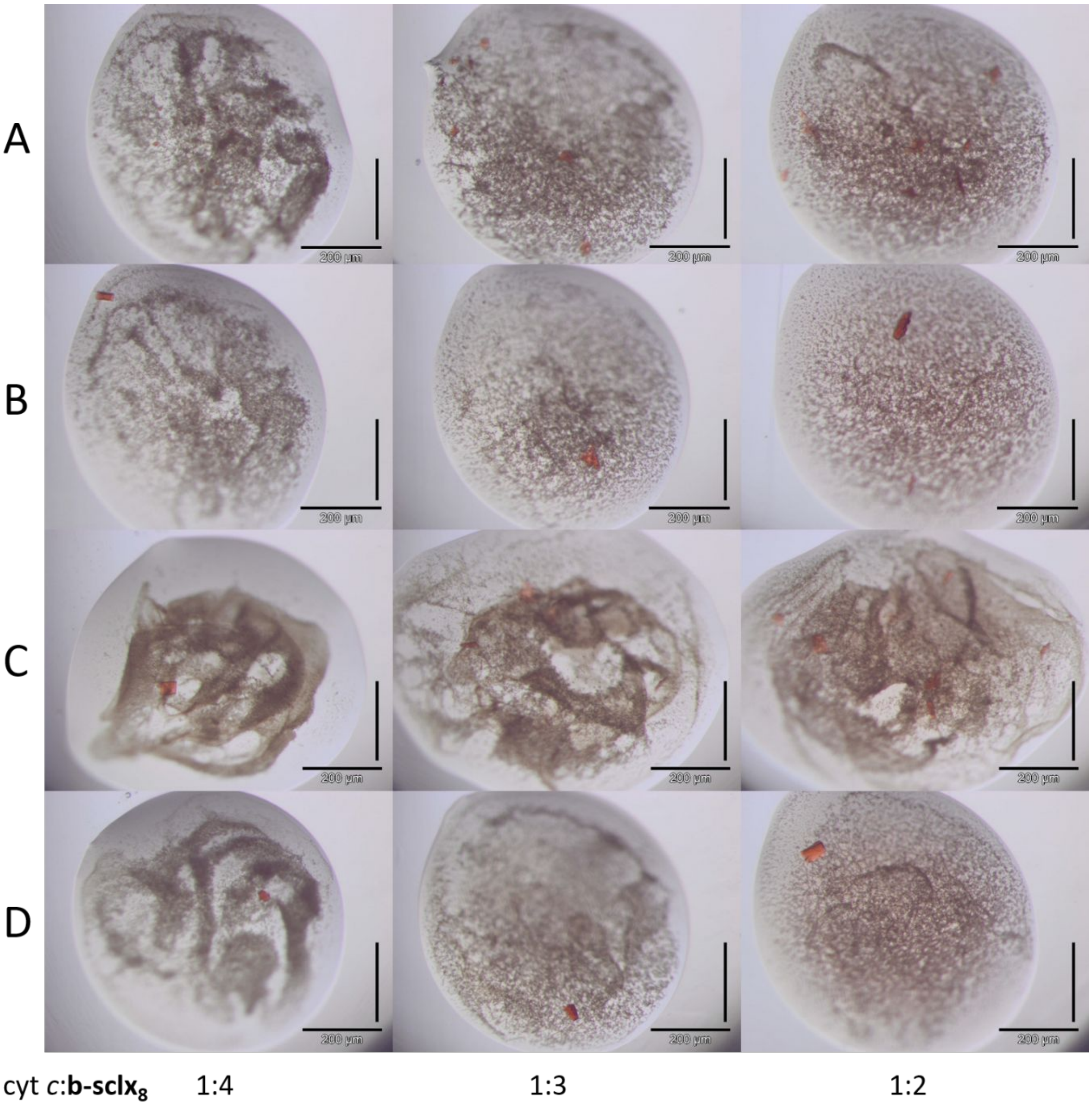

Figure S1: Microscope images of co-crystallization drops containing cyt *c* and 2-4 eq. **b-sclx$_8$**. The conditions contained 20 % PEG 3350 and 200 mM **(A)** magnesium formate, **(B)** ammonium formate, **(C)** ammonium chloride and **(D)** potassium formate. These conditions were selected based on (Jena JCSG++ HTS conditions A5, A8, A9, A10, A12, G8, G9, G10). Crystals of < 40 µm dimension are evident amid heavy, brown precipitate. The scale bar is 200 µm.

Table S1. Crystallization conditions, X-ray data collection, processing and refinement statistics.[a]

Crystallization Conditions	
Protein (mM)	0.75
**b-sclx$_8$** (mM)	2
PEG 3350 (%)	20
Ammonium formate (mM)	200
Data Collection	
Light Source	SOLEIL, PROXIMA-2A
Wavelength (Å)	0.98009
Space group	*C*121
Cell constants (Å, °)	120.9 70.2, 70.2<br>90.0, 102.9, 90.0
Resolution (Å)	50.6 - 3.0 (3.1 - 3.0)
# reflections	73389 (3266)
# unique reflections	10928 (561)
Multiplicity	6.7 (5.8)
I/σ (I)	8.9 (2.6)
Completeness (%)	96.3 (100.0)
R_{meas}[b] (%)	24.1 (128.6)
R_{pim}[c] (%)	9.3 (52.6)
$CC_{1/2}$	98.9 (88.2)
Solvent content (%)	52
Refinement	
R_{work} (%)	23.3
R_{free} (%)	26.1
rmsd bonds (Å)	0.005
rmsd angles (°)	0.65
# molecules in asymmetric unit	
Protein	4
**b-sclx$_8$**	3
PEG	1
water	41
Average B-factor (Å^2)	75.3
Clashscore	1.3
Ramachandran analysis, % residues in,[d]	
favoured regions	99.1
allowed regions	0.95
PDB code	7BBT

[a]Values in parentheses correspond to the highest resolution shell; [b]$R_{meas} = \sum_{hkl} \surd(n/n\text{-}1) \sum_i |I_i(hkl) - \langle I(hkl) \rangle| / \sum_{hkl} \sum_i I_i(hkl)$; [c]$R_{pim} = \sum_{hkl} \surd(1/n\text{-}1) \sum^{n}_{i=1} |Ii(hkl) - \langle I(hkl) \rangle| / \sum_{hkl} \sum_i I_i(hkl)$; [d]Calculated in MolProbity

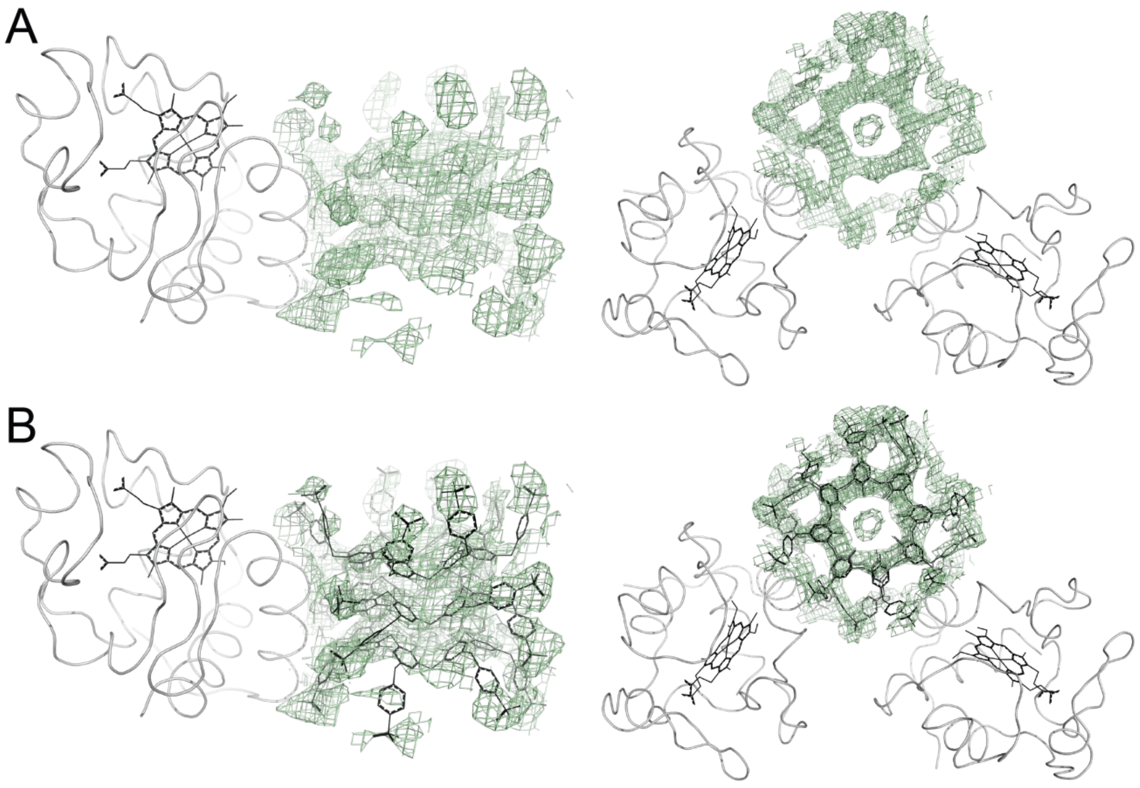

Figure S2: The presence of the trimeric **b-sclx$_8$** stack was clear in the unbiased electron density maps, contoured at 0.5 σ (green mesh). **(A)** Without and **(B)** with the calixarene model. Left and right images are related by a 90° rotation.

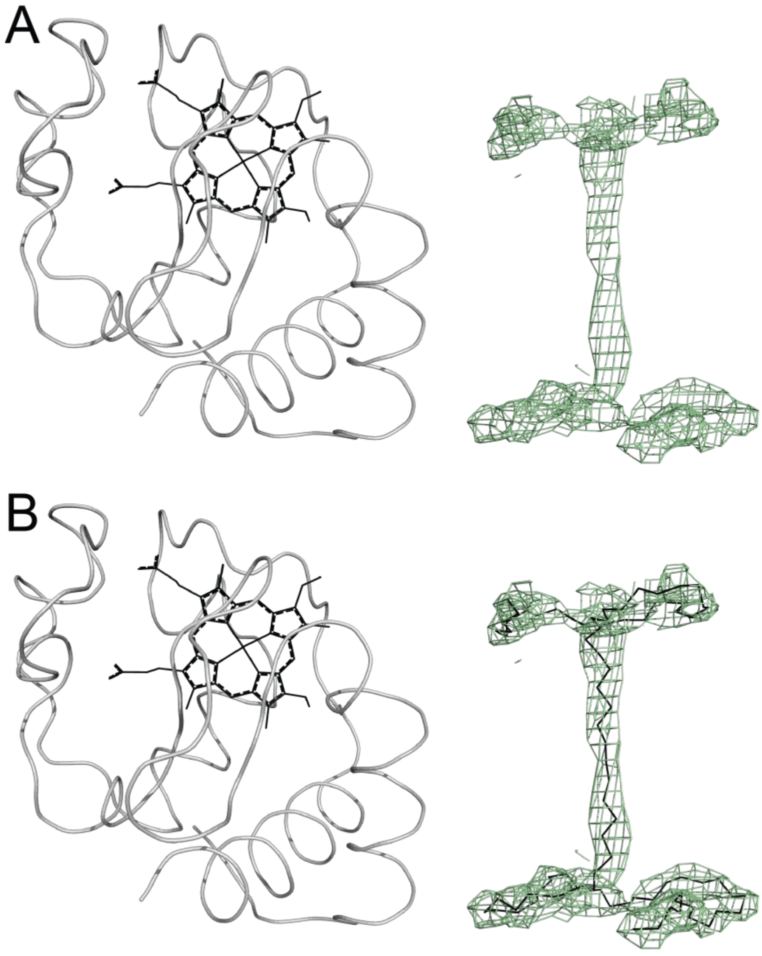

Figure S3: The presence of PEG in the unbiased electron density maps contoured at 0.2 σ (green mesh). **(A)** Without and **(B)** with the PEG model.

References

1. Olteanu, A.; Patel, C. N.; Dedmon, M. M.; Kennedy, S.; Linhoff, M. W.; Minder, C. M.; Potts, P. R.; Deshmukh, M.; Pielak, G. J. Stability and apoptotic activity of recombinant human cytochrome *c*. *Biochem. Biophys. Res. Commun.* **2003**, *312*, 733-740.
2. Volkov, A. N.; Vanwetswinkel, S.; Van de Water, K.; van Nuland, N. A. J. Redox-dependent conformational changes in eukaryotic cytochromes revealed by paramagnetic NMR spectroscopy. *J. Biomol. NMR* **2012**, *52*, 245-256.
3. McGovern, R. E.; Fernandes, H.; Khan, A. R.; Power, N. P.; Crowley, P. B. Protein camouflage in cytochrome *c*-calixarene complexes. *Nat. Chem.* **2012**, *4*, 527-533.
4. Hubble, L. J.; Clark, T. E.; Makha, M.; Raston, C. L. Selective diameter uptake of single-walled carbon nanotubes in water using phosphonated calixarenes and 'extended arm' sulfonated calixarenes. *J. Mater. Chem.* **2008,** *18*, 5961-5966.
5. Delaglio, F.; Grzesiek, S.; Vuister, G. W.; Zhu, G.; Pfeifer, J.; Bax, A. NMRPipe: a multidimensional spectral processing system based on UNIX pipes*. J. Biomol. NMR* **1995**, *6*, 277-293.
6. Vranken, W. F.; Boucher, W.; Stevens, T. J.; Fogh, R. H.; Pajon, A.; Llinas, M.; Ulrich, E. L.; Markley, J. L.; Ionides, J.; Laue, E. D. The CCPN data model for NMR spectroscopy: development of a software pipeline. *Proteins*, **2005**, *59*, 687-696.
7. Vonrhein, C.; Flensburg, C.; Keller, P.; Sharff, A.; Smart, O.; Paciorek, W.; Womack, T.; Bricogne, G. Data processing and analysis with the autoPROC toolbox. *Acta Crystallogr., Sect. D: Biol. Crystallogr*. **2011**, *67*, 293-302.
8. Kabsch, W. XDS. *Acta Crystallogr., Sect. D: Biol. Crystallogr.* **2010**, *66*, 125
9. Evans, P. R.; Murshudov, G. N. How good are my data and what is the resolution? Acta Crystallogr., *Sect. D: Biol. Crystallogr.* **2013**, *69*, 1204-1214.
10. Evans, P. R. An Introduction to Data Reduction: Space-Group Determination, Scaling and Intensity Statistics. *Acta Crystallogr., Sect. D: Biol. Crystallogr.* **2011**, *67*, 282−292.
11. McCoy, A. J.; Grosse-Kunstleve, R. W.; Adams, P. D.; Winn, M. D.; Storoni, L. C.; Read, R. J. Phaser crystallographic software. *J. Appl. Crystallogr.* **2007**, *40*, 658-674.
12. Engilberge, S.; Rennie, M. L.; Dumont, E.; Crowley, P. B. Tuning protein frameworks via auxiliary supramolecular interactions. *ACS Nano* **2019**, *13*, 10343-10350.
13. Macrae, C. F.; Bruno, I. J.; Chisholm, J. A.; Edgington, P. R.; McCabe, P.; Pidcock, E.; Rodriguez-Monge, L.; Taylor, R.; Streek, J.; Wood, P. A. Mercury CSD 2.0–new features for the visualization and investigation of crystal structures. *J. Appl. Crystallogr.* **2008**, *41*, 466-470.

14. Smart, O. S.; Womack, T. O.; Sharff, A.; Flensburg, C.; Keller, P.; Paciorek, W.; Vonrhein, C.; Bricogne, G. *grade*, version 1.2.2; Global Phasing Limited: Cambridge, U.K., 2011

15. Emsley, P.; Cowtan, K. Coot: model-building tools for molecular graphics. *Acta Crystallogr., Sect. D: Biol. Crystallogr.* **2004**, *60*, 2126-2132.

16. Adams, P. D.; Afonine, P. V.; Bunkoczi, G.; Chen, V. B.; Davis, I. W.; Echols, N.; Headd, J. J.; Hung, L. W.; Kapral, G. J.; GrosseKunstleve, R. W.; McCoy, A. J.; Moriarty, N. W.; Oeffner, R.; Read, R. J.; Richardson, D. C.; Richardson, J. S.; Terwilliger, T. C.; Zwart, P. H. PHENIX: A Comprehensive Python-Based System for Macromolecular Structure Solution. *Acta Crystallogr., Sect. D: Biol. Crystallogr.* **2010**, *66*, 213-221.

17. Bricogne, G.; Blanc, E.; Brandl, M.; Flensburg, C.; Keller, P.; Paciorek, W.; Roversi, P.; Sharff, A.; Smart, O. S.; Vonrhein, C.; Womack, T. O. *BUSTER*, Version 2.10.3; Global Phasing, Ltd., Cambridge, U.K., 2017.

18. Alex, J. M.; Rennie, M. L.; Volpi, S.; Sansone, F.; Casnati, A.; Crowley, P. B. Phosphonated calixarene as a “molecular glue” for protein crystallization. *Cryst. Growth Des*. **2018**, *18*, 2467-2473.

19. Williams, C. J.; Headd, J. J.; Moriarty, N. W.; Prisant, M. G.; Videau, L. L.; Deis, L. N.; Verma, V.; Keedy, D. A.; Hintze, B. J.; Chen, V. B.; Jain, S.; Lewis, S. M.; Arendall, W. B.; Snoeyink, J.; Adams, P. D.; Lovell, S. C.; Richardson, J. S.; Richardson, D. C. MolProbity: More and Better Reference Data for Improved All-Atom Structure Validation. *Protein Sci*. **2018**, *27*, 293−315.

20. Collaborative Computational Project, N. The CCP4 suite: programs for protein crystallography. *Acta Crystallogr., Sect. D: Biol. Crystallogr.* **1994**, *50*, 760-763.
